# Cleavage of Syndecan-1 Promotes the Proliferation of the Basal-Like Breast Cancer Cell Line BT-549 Via Akt SUMOylation

**DOI:** 10.3389/fcell.2021.659428

**Published:** 2021-05-25

**Authors:** Satomi Nadanaka, Yaqiang Bai, Hiroshi Kitagawa

**Affiliations:** Laboratory of Biochemistry, Kobe Pharmaceutical University, Kobe, Japan

**Keywords:** proteoglycan, chondroitin sulfate, breast cancer, syndecan-1, SUMOylation

## Abstract

Basal-like breast cancer is characterized by an aggressive clinical outcome and presence of metastasis, for which effective therapies are unavailable. We have previously shown that chondroitin 4-*O*-sulfotransferase-1 (C4ST-1) controls the invasive properties of the basal-like breast cancer cell line BT-549 by inducing matrix metalloproteinase (MMP) expression through the N-cadherin/β-catenin pathway. Here we report that C4ST-1 controls the proliferation of BT-549 cells via the MMP-dependent cleavage of syndecan-1. Syndecan-1 is a membrane-bound proteoglycan associated with an aggressive phenotype and poor prognosis in breast cancer. In addition, the cleavage of syndecan-1 at a specific juxtamembrane cleavage site is implicated in the pathophysiological response in breast cancer. Knockout of C4ST-1 remarkably suppressed both the cleavage of syndecan-1 and proliferation of BT-549 cells. Kinases (AKT1, ERK1/2, PI3K, and STAT3) comprising cancer proliferative pathways are phosphorylated in C4ST-1 knockout cells at a level similar to that in parental BT-549 cells, whereas levels of phosphorylated S6 kinase and SUMOylated AKT (hyperactivated AKT observed in breast cancer) decreased in C4ST-1 knockout cells. An MMP inhibitor, GM6001, suppressed the small ubiquitin-like modifier (SUMO) modification of AKT, suggesting that cleavage of syndecan-1 by MMPs is involved in the SUMO modification of AKT. Forced expression of the cytoplasmic domain of syndecan-1, which is generated by MMP-dependent cleavage, increased the SUMO modification of AKT and global protein SUMOylation. Furthermore, syndecan-1 C-terminal domain-expressing BT-549 cells were more proliferative and sensitive to a potent SUMOylation inhibitor, tannic acid, compared with BT-549 cells transfected with an empty expression vector. These findings assign new functions to the C-terminal fragment of syndecan-1 generated by MMP-dependent proteolysis, thereby broadening our understanding of their physiological importance and implying that the therapeutic inhibition of syndecan-1 cleavage could affect the progression of basal-like breast cancer.

## Introduction

Chondroitin sulfate (CS), a glycosaminoglycan (GAG), is present on the cell surface and in the extracellular matrix (Sugahara et al., 2003). There is ample evidence for the pro-tumorigenic role of CS in the enhancement of cell proliferation, cell motility, and metastasis. CS is a linear sulfated polymer consisting of repeating disaccharide units of glucuronic acid (GlcUA) and *N*-acetylgalactosamine (GalNAc) [-GlcUA-GalNAc-]*_*n*_*. During the synthesis of the CS backbone, various sulfotransferases catalyze sulfation. Chondroitin-4-*O*-sulfotransferase (C4ST-1) is involved in the biosynthesis of A-units [GlcUA-GalNAc(4-*O*-sulfate)] and E-disaccharide units [GlcUA-GalNAc(4,6-*O*-disulfates)] (Nadanaka et al., 2008; Mikami and Kitagawa, 2013; Nadanaka et al., 2020). Specific sulfation patterns are believed to underlie the distinct functional roles of CS not only under physiological conditions but also in tumor development and progression (Nadanaka et al., 2018).

Previously, C4ST-1 (Gene symbol: CHST11) was shown to be upregulated in breast cancer cells (Iida et al., 2015). In addition, the gene expression of *C4ST-1* has been correlated with the progression of breast cancer (Hazan et al., 2000). Most recently, Behrens et al. (2020) reported a new role of C4ST-1 in the induction of epithelial–mesenchymal transition and stem cell-like properties in breast cancer. These aggressive tumor phenotypes are thought to be exerted via protein–sugar interactions that are defined by specific sulfation patterns generated by C4ST-1. A few binding partners of CS produced by C4ST-1 have been identified. CS produced by C4ST-1 functions as a P-selectin ligand in aggressive breast cancer cells (Cooney et al., 2011). In addition, we have previously reported that the binding of CS produced by C4ST-1 to N-cadherin triggers endocytosis-dependent activation of the N-cadherin/β-catenin pathway to enhance the metastatic properties of the basal-like breast cancer cell line BT-549 (Nadanaka et al., 2018). However, the molecular mechanism underlying the tumor-promoting functions induced by C4ST-1 is not completely understood.

We have shown that CS produced by C4ST-1 induces the expression of matrix metalloproteinase 9 (MMP9) through the activation of the N-cadherin/β-catenin pathway (Nadanaka et al., 2018). Binding of C4ST-1-synthesized CS to N-cadherin triggers endocytosis and proteolysis of N-cadherin. Further, the C-terminal domain of N-cadherin forms a complex with β-catenin is released and translocates into the nucleus, where the target genes such as MMP9 are transcriptionally induced by β-catenin. Increased expression of MMP9 enhances invasion activity of BT-549 cells. In contrast, C4ST-1 knockout decreases the β-catenin-dependent transcriptional induction of MMP9 and subsequently suppresses the enhanced invasion activity of BT-549 cells. Recently, we found that the proliferation of C4ST-1-knockout BT-549 cells was decreased compared with that of parental BT-549 cells. These results have raised the possibility that breast cancer cells acquire not only invasive properties but also proliferative capacity by taking advantage of MMP9. However, it remains unclear what substrate proteins are cleaved by MMP9 in BT-549 cells. Syndecan-1 (SDC1), a cell surface proteoglycan, is thought to serve as a promising substrate for MMPs (Manon-Jensen et al., 2013). SDC1 has been implicated in promoting breast cancer progression and is highly expressed in basal-like breast cancers (Rousseau et al., 2011; Nguyen et al., 2013; Sayyad et al., 2019). In addition, the cleavage of SDC1 by MMPs is involved in tumor invasion and proliferation (Su et al., 2008; Wang et al., 2014; Szatmari et al., 2015; Jang et al., 2020). These findings prompted us to examine whether the proliferation of BT-549 cells is controlled by the cleavage of SDC1 by MMPs. Here we examined how MMPs promote the proliferation of BT-549 cells through the proteolysis of SDC1.

## Materials and Methods

### Cell Culture and Stable Transfection

The human breast cancer cell line BT-549 (ATCC^®^ HTB-122^TM^), ER-, and ERBB2-negative (triple-negative and basal B subtype) breast cancer cell lines were obtained from American Type Culture Collection (ATCC) (Lacroix and Leclercq, 2004; Kao et al., 2009). The origin of BT-549 cells is “papillary, invasive ductal carcinoma,” a non-frequent type (Bambang et al., 2013). C4ST-1-knockout BT-549 cells were generated using Crispr-Cas9 genome editing system as described previously (Nadanaka et al., 2018). Both cells were cultured in RPMI 1640 supplemented with 10% heat-inactivated fetal bovine serum (FBS), 100 units/mL penicillin, 100 μg/mL streptomycin, and 1% L-glutamine. The expression plasmids [p3xFLAG-CMV-14, p3xFLAG-CMV-14-hSDC1 (full-length), and p3xFLAG-CMV-14-hSDC1 (Δ 29–245)] were transfected into BT-549 cells using Lipofectamine 3000 (Invitrogen) according to the manufacturer’s instructions. Transfectants were cultured in the presence of 25 μg/mL G418. Colonies surviving in the presence of 25 μg/mL G418 were collected and propagated for further experiments.

### Plasmid Construction

The human syndecan-1 (SDC1) gene (NM_001006946) was obtained from a HeLa cDNA library by polymerase chain reaction (PCR) using the following primers.

Forward primer for the amplification of full-length SDC1:

5′-CCATCGATGCCACC**ATG**AGGC-3′ (underline, *Cla*I site; bold, start codon).

Reverse primer for the amplification of full-length SDC1:

5′-GCTCTAGAGGCATAGAATTCCTCCTGTTTG-3′ (underline, *Xba*I site).

p3xFLAG-CMV-14-hSDC1 was constructed by inserting the *Cla*I-*Xba*I fragments of a PCR product into the *Cla*I and *Xba*I sites of p3xFLAG-CMV-14 (#E4901, Sigma-Aldrich, St. Louis, MO). An expression vector of the C-terminal fragment of SDC1, p3xFLAG-CMV-14-hSDC1 (CTF), was constructed by site-directed deletion mutagenesis by inverse PCR using the following primers and KOD One DNA polymerase (TOYOBO, Osaka, Japan). The human SDC1 core protein contains MMPs cleavage sites (Gly^82^ – Leu^83^, Glu^236^ – Gln^237^, and Gly^245^ – Leu^246^) (Manon-Jensen et al., 2013) and a transmembrane region (Val^252^ – Tyr^276^). Thus, the C-terminal fragment of SDC1 generated by MMPs (Leu^246^ – Tyr^309^) was expressed by fusing to the N-terminal region, including a signal peptide (Met^1^ – Leu^28^).

Forward primer for mutagenesis:

5′-CTCACAGGGCCTCCTGGACAGGAAAG-3′

Reverse primer for mutagenesis:

5′-ATTAGTAGCCACAATTTGCGGCAGGGC-3′

An expression vector of the N-terminal fragment of SDC1, p3xFLAG-CMV-14-hSDC1 (NTF), was constructed by site-directed deletion mutagenesis by inverse PCR using the following primers and KOD One DNA polymerase (TOYOBO, Osaka, Japan).

Forward primer for mutagenesis:

5′-ACTAGAGGATCCCGGGCTGAC-3′

Reverse primer for mutagenesis:

5′-GCCGGTGGGTTCTGGAGACG-3′

Integrity of the resulting plasmids was confirmed by sequencing the entire coding region and the ligation joints.

### Proliferation Assay

Cells were plated on 24-well plates at 5,000 cells/well and cultured for 3 days in the presence or absence of 5 munits/well of chondroitinase ABC (Chase ABC) (Seikagaku Biobusinesses, Tokyo, Japan) or 20 μM GM6001 (MMP inhibitor, #BML-EI300-0001, Enzo, Famingdale, NY, United States). To achieve exhaustive digestion of cellular CS chains, cells were digested in the serum-free media ASF Medium 104 (Ajinomoto Co., Inc., Tokyo, Japan) for 4 days, and 5 munits/well of chondroitinase ABC was added twice at 0 and 2 days. Anti-chondroitin sulfate antibody (clone 2B6) (#PRPG-BC-M02, Cosmo Bio Co., Ltd., Tokyo, Japan) was used to confirm chondroitinase ABC activity. After the cells were washed with phosphate-buffered saline (PBS), cellular lactate dehydrogenase was released from cells by adding 200 μL of lysis buffer containing 0.18% (w/v) Triton X-100. The enzyme activity of cellular lactate dehydrogenase was measured as an indicator of cell viability using the CytoTox-ONE^TM^ Homogeneous Membrane Integrity Assay (Promega, Madison, WI, United States). To examine the proliferative pathways of BT-549 cells, GSK690693 (AKT inhibitor, #S1113, Selleck Chemicals, Houston, TX, United States) and tannic acid (SUMOylation inhibitor, #403040, Sigma-Aldrich, St. Louis, MO, United States) were used at indicated concentrations.

For determining the growth curve, cells were seeded on a 6-cm dish at a concentration of 2 × 10^4^ cells/dish and cultured for 0, 1, 2, 4, 6, 8, 10, 13, and 15 days. The cells were harvested, stained with crystal violet-citric acid staining solution, and counted using a hemocytometer.

### Clonogenic Assay

Clonogenic assay was performed according to a method described previously (Kawamura et al., 2013). In brief, BT-549 cells were trypsinized to obtain single cell suspensions and seeded at a density of 50 cells/well in 6-well plates. The cells were then cultured for 9 days, fixed, and stained with crystal violet. Colonies consisting of more than 50 cells were counted.

### Flow Cytometry

Cells (1 × 10^5^ cells) treated with or without 25 μM GM6001 for 4 days were fixed with PBS containing 4% paraformaldehyde on ice for 30 min. After washing with PBS, the cells were incubated with PBS containing 2% BSA on ice for 30 min, and then stained with the anti-SDC1 antibody (dilution ratio 1:200) (HPA006185, Sigma-Aldrich) on ice. After 2 h, the cells were washed and incubated with rabbit IgG antibody conjugated with Alexa 488 (dilution ratio 1:200) on ice for 1 h. The cells were analyzed using a BD Accuri^TM^ C6 flow cytometer (BD Biosciences, San Jose, CA, United States).

### Immunoblotting

For detecting the MMP-dependent cleavage of SDC1, the cells were cultured in the presence or absence of 25μM GM6001 for 4 days. The medium conditioned for 4 days by the cells was collected and centrifuged at 13,000 rpm for 10 min. Supernatants were incubated with 10 μL of protein G-Sepharose beads for 2 h at 4°C to remove serum immunoglobulin. After centrifugation at 4,000 rpm for 2 min, the resulting supernatants were subject to immunoblotting. The cells were washed with PBS and solubilized with M-PER (Thermo Fisher Scientific, Waltham, MA, United States) containing a protease inhibitor cocktail (Nacalai Tesque, Kyoto, Japan) for 30 min on ice. Cell lysates were centrifuged at 13,500 rpm for 10 min, and the resulting supernatants were analyzed by immunoblotting. For detecting SDC1, the supernatants were digested with or without 0.5 munits of heparitinase (HSase), 0.5 munits of heparinase (Hepase), and 1 munits of Chase ABC for 30 min at 37°C. All these glycosaminoglycan lyases (GAGase) were purchased from Seikagaku Biobusinesses (Tokyo, Japan).

For analysis of signaling pathways, the cells were solubilized with lysis buffer [1% Triton X-100, 20 mM Tris-HCl (pH 7.5), 0.15 M NaCl, 1 mM EDTA, 10% glycerol, 10 μM MG132, protease and phosphatase inhibitor cocktail (Nacalai Tesque, Kyoto, Japan)] for 30 min on ice. The protein concentration of each sample was determined by Pierce^TM^ BCA Protein Assay Kit (Thermo Fisher Scientific).

To detect the SUMOylated AKT, the cells were solubilized with M-PER (10 μM MG132, protease and a phosphatase inhibitor cocktail, and 4 mM *N*-ethylmaleimide) for 30 min on ice, and cell lysates were centrifuged at 13,000 rpm for 10 min. Supernatants were incubated with anti-phospho-AKT (Ser473) antibody (dilution ratio 1:500) (#9271, Cell Signaling Technology, Danvers, MA) or anti-AKT antibody (dilution ratio 1:500) (#9272, Cell Signaling Technology, Danvers, MA) at 4°C overnight. After adding 10 μL of Protein G-Sepharose, each sample was incubated for 2 h at 4°C. The Protein G-Sepharose beads recovered by centrifugation were washed with lysis buffer.

To analyze the SUMOylated proteins, SUMO-QAPTURE-T^®^ kit (#BML-UW1000A, Enzo Famingdale, NY, United States) was used. Each sample was resolved on Bullet PAGE One precast gels (5–15%), transferred to polyvinylidene fluoride membranes, and incubated overnight with the antibodies mentioned in Supplementary Table 1.

### Knockdown of MMP2, 7, 9, and 14 by siRNA

Cells were transfected with the following siRNAs corresponding to MMP2, MMP7, MMP9, and MMP14. Silencer^®^ Select siMMP2 (Cat. #4427038, Assay ID: s8852), siMMP7 (Cat. #4427037, Assay ID: s8858), siMMP14 (Cat. #4427038, Assay ID: s8878), and MMP9 siRNA (Cat. # sc-29400) were purchased from Thermo Fisher Scientific and Santa Cruz Biotechnology, Inc., respectively. Cells were transfected with 5 nM siRNA(MMP2, MMP7, and MMP14) or 25 nM (MMP9) using Lipofectamine 2000 (Thermo Fisher Scientific) according to the manufacturer’s protocol and cultured for 2 days. Each siRNA-induced gene knockdown was assessed by real-time PCR.

Total RNA was isolated from the cells using the Maxwell^®^ 16 LEV simplyRNA purification kit (Promega). For reverse transcription, 1 μg of total RNA was treated with Moloney murine leukemia virus reverse transcriptase (Invitrogen) using random primers [non-adeoxyribonucleotide mixture; pd(N)_9_] (Takara Bio Inc., Shiga, Japan). Quantitative real-time PCR was performed using FastStart DNA Master Plus SYBR Green I in LightCycler^®^ 96 (Roche Applied Science) according to the manufacturer’s protocols. The housekeeping gene *G3PDH* was used as an internal control for quantification. The primers used for real-time PCR are mentioned in Supplementary Table 2.

### Statistics and Reproducibility

Data are expressed as the mean ± standard deviation of the mean. Statistical significance was determined using the Tukey–Kramer multiple comparison method and Student’s *t*-test. Statistical analyses were performed using KaleidaGraph version 4.5.1. For all analyses, *n* = number of experiments and differences were considered statistically significant at a *p*-value of <0.05. All experiments were reproduced with similar results a minimum of three times, and the exact number of repetitions is provided in the figure legends.

## Results

### Deficiency for C4ST-1 Suppresses the Proliferation of BT-549 Cells

**FIGURE 1:**
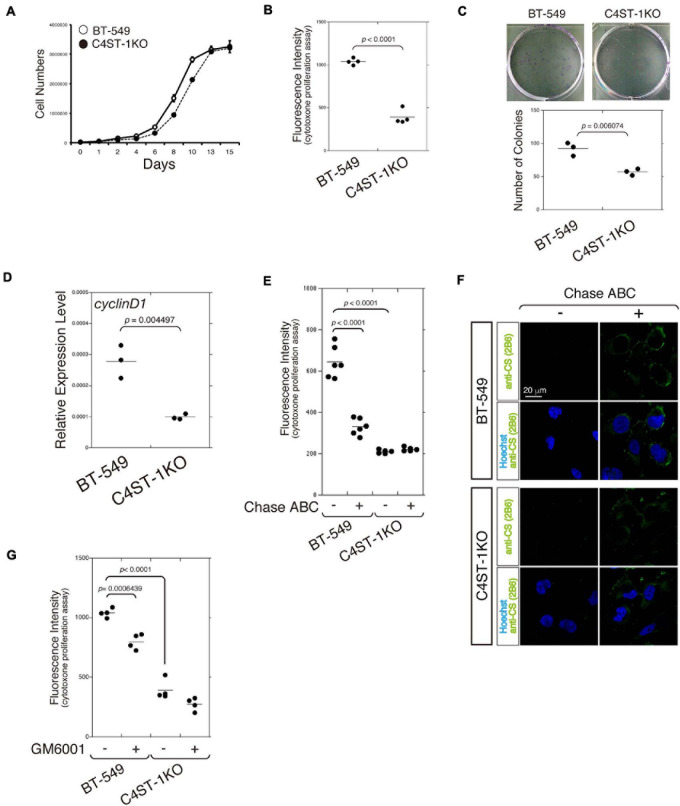
C4ST-1 deficiency suppresses the proliferation of BT-549 cells. **(A)** Growth curves of parental BT-549 and C4ST-1 KO cells. **(B)** Proliferation of BT-549 (*n* = 4) and C4ST-1 KO cells (*n* = 4) was measured by CytoTox-ONE^TM^ Assay. **(C)** Proliferation of BT-549 and C4ST-1 KO cells was examined by colony formation assay. Both the cell types were seeded at a concentration of 50 cells/well in 6-well plate and cultured for 9 days. The colonies were stained with crystal violet, and observed under a light microscope (Left). The number of colonies of BT-549 (*n* = 3) and C4ST-1 KO cells (*n* = 3) was compared (Right). **(D)** The level of cyclin D1 in BT-549 and C4ST-1 KO cells was measured by real-time PCR (*n* = 3 each). **(E)** Proliferation of BT-549 (*n* = 4) and C4ST-1 KO cells (*n* = 4) treated with or without Chase ABC was examined by CytoTox-ONE^TM^ Assay. Cells were digested in the serum-free medium ASF Medium 104 for 4 days by adding 5 munits/well of Chase ABC twice at 0 and 2 days. **(F)** Cells digested with or without Chase ABC were stained by anti-CS antibody (clone 2B6), which detects the terminal unsaturated disaccharide of CS chains generated by Chase ABC. **(G)** Proliferation of BT-549 (*n* = 4) and C4ST-1 KO cells (*n* = 4) treated with or without GM6001 was measured. Statistical significance was determined using Student’s *t*-test. Statistical analyses were performed using KaleidaGraph version 4.5.1.

The cell growth curve was plotted to compare the time-dependent increase in cell number between *C4ST-1* knockout cells (C4ST-1 KO cells) and parental BT-549 cells (Figure 1A). In addition, cell growth of these two cell lines was measured using the CytoTox-ONE^TM^ cell growth assay kit (Figure 1B). Furthermore, the ability of cells in culture to grow and divide into groups was assessed by the colony formation assay (Figure 1C). These results indicate that cell growth was remarkably suppressed by the loss of *C4ST-1* expression. We next examined the expression of *cyclin D1*, which has been reported as the target of β-catenin because a previous study showed that C4ST-1 upregulates β-catenin-dependent transcriptional activities (Nadanaka et al., 2018). Deficiency of C4ST-1 significantly decreased the expression of *cyclin D1* in C4ST-1 KO cells than in parental BT-549 cells (Figure 1D). We examined the effect of CS removal by digestion with Chase ABC on the proliferation of these two cell lines (Figure 1E). The proliferation of BT-549 cells was significantly attenuated by digestion with Chase ABC. The effect of Chase ABC on cellular CS chains was confirmed using anti-CS antibody (clone 2B6), which recognizes unsaturated disaccharide neoepitopes generated after the digestion of CS chains by Chase ABC (Figure 1F). We next investigated whether MMPs are involved in the proliferation of BT-549 cells (Figure 1G) because the loss of *C4ST-1* expression decreases the expression of *MMP9*, which is a target gene of β-catenin (Nadanaka et al., 2018). The proliferation of BT-549 cells was significantly inhibited by treatment with GM6001 (an MMP inhibitor), whereas C4ST-1 KO cells were not affected. These results suggest that MMPs play an important role in C4ST-1-dependent proliferation of BT-549 cells.

### Loss of *C4ST-1* Expression Suppresses the MMP-Mediated Cleavage of SDC1

**FIGURE 2:**
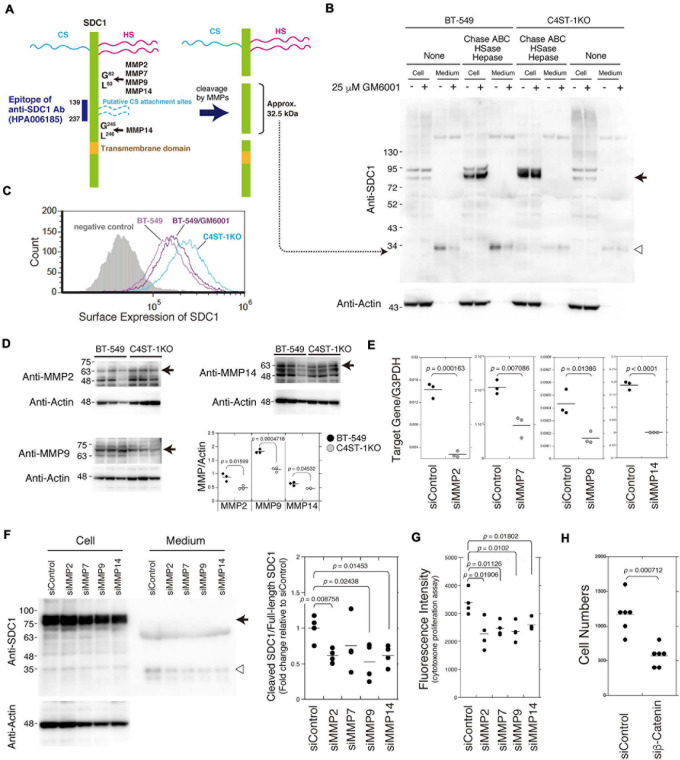
Inhibition of the cleavage of SDC1 by loss of *C4ST-1* expression. **(A)** Schematic representation of the cleavage sites of MMPs, HS/CS attachment sites on SDC1, and the epitope of the anti-SDC1 antibody used in this study. CS chains are indicated by dashed lines because Ser^206^ and Ser^216^ are not modified with CS chains in BT-549 cells. In addition, CS chains are attached to Ser^37^, Ser^45^, or Ser^47^, because a SDC1 core protein is modified with CS chains according to our previous study (Nadanaka et al., 2018). Anti-SDC1 antibody used in this study recognizes the SDC1 fragment (approx. 32.5 kDa) after cleavage by MMPs. **(B)** BT-549 and C4ST-1 KO cells were incubated in the presence (+) or absence (−) of GM6001. Cell lysates and conditioned medium prepared from each cell were treated with (+) or without (−) Chase ABC, HSase, and Hepase, and then subject to immunoblotting using the anti-SDC1 antibody. Arrow and open triangle indicate the core protein of full-length SDC1 and the SDC1 fragment after cleavage by MMPs, respectively. **(C)** The surface expression of SDC1 in BT-549 and BT-549 treated with GM6001, and C4ST-1KO cells were examined by flow cytometry. **(D)** The expression level of MMP2, MMP9, and MMP14 in BT-549 and C4ST-1KO cells was examined by immunoblotting. **(E)** The levels of MMP2, MMP7, MMP9, and MMP14 in BT-549 cells transfected either with si-Control or si-MMP2, 7, 9, or 14 were measured by real-time PCR. **(F)** The effect of knockdown of MMP2, 7, 9, or 14 on the cleavage of SDC1 was examined. Arrow and open triangle indicate the core protein of full-length SDC1 and the SDC1 fragment after cleavage by MMPs, respectively. At the right side, the ratio of cleaved SDC1 to full-length SDC1 is shown as fold change relative to that of siControl. **(G)** The effect of knockdown of MMP2, MMP7, MMP9, and MMP14 on the proliferation of BT-549 cells (*n* = 4, each) was examined by CytoTox-ONE^TM^ Assay. **(H)** The effect of knockdown of β-catenin on the proliferation of BT-549 cells (*n* = 6) was investigated. Statistical significance was determined using Student’s *t*-test.

As shown in Figure 2A, SDC1 is a hybrid-type transmembrane proteoglycan that consists of both CS and heparan sulfate chains. MMP cleavage sites and the HS (Ser^37^, Ser^45^, and Ser^47^) and CS attachment sites (Ser^206^ and Ser^216^) on SDC1 have been reported in a previous study (Manon-Jensen et al., 2013). These MMP cleavage sites and HS/CS attachment sites are shown in Figure 2A. Full-length SDC1 core proteins were detected in the cell lysates after digestion in combination with Chase ABC, HSase, and Hepase (Figure 2B, arrow). It is estimated that the SDC1 fragments generated by MMPs provide a band of approximately 32.5 kDa because the anti-SDC1 antibody developed against amino acids 139–237 was used in this study (Figure 2A). The approximately 32.5 kDa band was detected in the BT-549 cell-conditioned medium digested with and without Chase ABC (Figure 2B, open triangle), suggesting that Ser^206^ and Ser^216^ are not modified with CS chains in BT-549 cells. Our previous study showed that CS chains are attached to a SDC1 core protein (Nadanaka et al., 2018), suggesting that Ser^37^, Ser^45^, or Ser^47^ can be modified with CS chains. This 32.5 kDa band disappeared after treating BT-549 cells with GM6001. These results suggest that the MMP-mediated proteolysis of SDC1 occurs in BT-549 cells. On the other hand, the cleavage of SDC1 by MMPs was strongly inhibited in C4ST-1 KO cells, suggesting that C4ST-1 controls MMP-mediated cleavage of SDC1.

We next compared the surface expression of SDC1 among BT-549 cells, GM6001-treated BT-549 cells, and C4ST-1 KO cells (Figure 2C). Consistent with the results shown in Figure 2B, the surface expression of SDC1 in C4ST-1 KO cells was higher than that in BT-549 cells. In addition, treatment of BT-549 cells with GM6001 increased the surface expression of SDC1.

A previous study reported that MMP2, MMP7, MMP9, and MMP14 are involved in the proteolysis of SDC1 (Manon-Jensen et al., 2013). In addition, all these MMPs are transcriptionally regulated by β-catenin. Thus, we confirmed that these MMPs are implicated in the cleavage of SDC1. We first compared the expression level of MMP2, MMP9, and MMP14 between BT-549 and C4ST-1KO cells (Figure 2D). The expression levels of three MMPs were significantly lower in C4ST-1KO cells than in BT-549 cells. Knockdown of each MMP suppressed the cleavage of SDC1 (Figures 2E,F) and attenuated the proliferation of BT-549 cells (Figure 2G). These results suggest that MMP-dependent proteolysis of SDC1 is associated with the proliferation of BT-549 cells. Furthermore, knockdown of β-catenin had a stronger effect on the proliferation of BT-549 cells (Figure 2H), implying that these four MMPs act on the cleavage of SDC1 in various combinations.

### Knockout of *C4ST-1* Downregulates the Proliferation of BT-549 Cells Via SUMOylation of AKT

**FIGURE 3:**
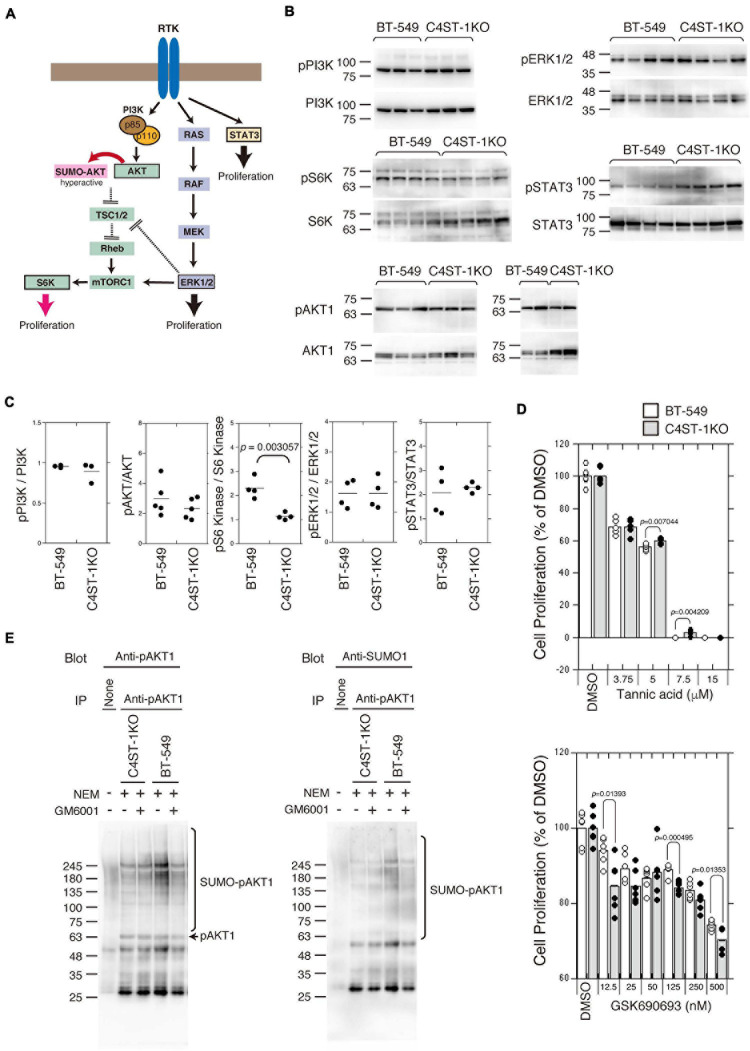
Suppression of SUMOylation of AKT1 by the loss of *C4ST-1* expression. **(A)** Some of the signaling pathways involved in cancer proliferation examined in this study are shown. **(B)** Phosphorylation of PI3K, AKT1, S6K, ERK1/2, and STAT3 in BT-549 and C4ST-1 KO cells was examined by immunoblotting using phospho-specific antibodies and total antibodies. **(C)** Phosphorylation of PI3K (*n* = 3), AKT1 (*n* = 5), S6K (*n* = 4), ERK1/2 (*n* = 4), and STAT3 (*n* = 4) in BT-549 and C4ST-1 KO cells was quantified by calculating the ratios of phosphorylated to total protein. **(D)** The effect of tannic acid and AKT inhibitor (GSK690693) on the proliferation of BT-549 and C4ST-1KO cells. **(E)** SUMOylation of AKT1 in BT-549 and C4ST-1 KO cells were examined. Both the cells were treated with (+) or without (−) GM6001, and lysed in the absence (−) or presence (+) of *N*-ethylmaleimide (NEM), which inhibits SUMO proteases. Immunoprecipitated phospho-AKT1 proteins were subject to immunoblotting using anti-pAkt1 and anti-SUMO1 antibodies.

Several signaling pathways involved in the proliferation and survival of breast cancer cells are illustrated in Figure 3A. We examined the activation of the key oncogenic protein kinases (PI3K, AKT1, ERK1/2, and STAT3) of each signaling pathway in BT-549 and C4ST-1 KO cells (Figures 3B,C). The phosphorylation levels of PI3K, AKT, ERK, and STAT3 were not affected by the loss of *C4ST-1* expression. The activation of the AKT downstream kinase, S6 kinase (S6K), was inhibited in C4ST-1 KO cells (Figures 3B,C). AKT is post-translationally modified by phosphorylation as well as the small ubiquitin-like modifier (SUMO) protein. SUMO modification regulates AKT activity and cell proliferation, and cancer progression is enhanced via the SUMOylation-mediated signaling pathway. Therefore, we examined whether AKT1 SUMOylation is affected by the loss of C4ST-1. Blockage of SUMOylation by tannic acid, an inhibitor of SUMOylation, strongly inhibited the proliferation of BT-549 and C4ST-1 KO cells at concentrations above 7.5 μM (Figure 3D). Of note, compared with BT-549 cells, C4ST-1 KO cells showed slight but significant resistance to 5 and 7.5 μM tannic acid. In addition, the proliferation of BT-549 cells was inhibited by the AKT inhibitor GSK690693; moreover, BT-549 cells were more resistant to GSK690693 than C4ST-1KO cells (Figure 3D). We next investigated the SUMO modification of AKT1 in BT-549 and C4ST-1 KO cells (Figure 3E). SUMO modification of phosphorylated AKT1 (pAKT1) was decreased in C4ST-1 KO cells than in BT-549 cells. In addition, treatment with GM6001 inhibited the SUMOylation of pAKT1. These results suggest that C4ST-1 regulates the SUMO modification of AKT1 mediated by MMPs. SUMOylation of AKT1 by C4ST-1 is, however, only one of many mechanisms of controlling cell proliferation in BT-549 cells because cell proliferation of C4ST-1KO cells was inhibited by tannic acid.

### C-Terminal Fragment of SDC1 Generated by MMPs Induces SUMO Modification of AKT1

**FIGURE 4:**
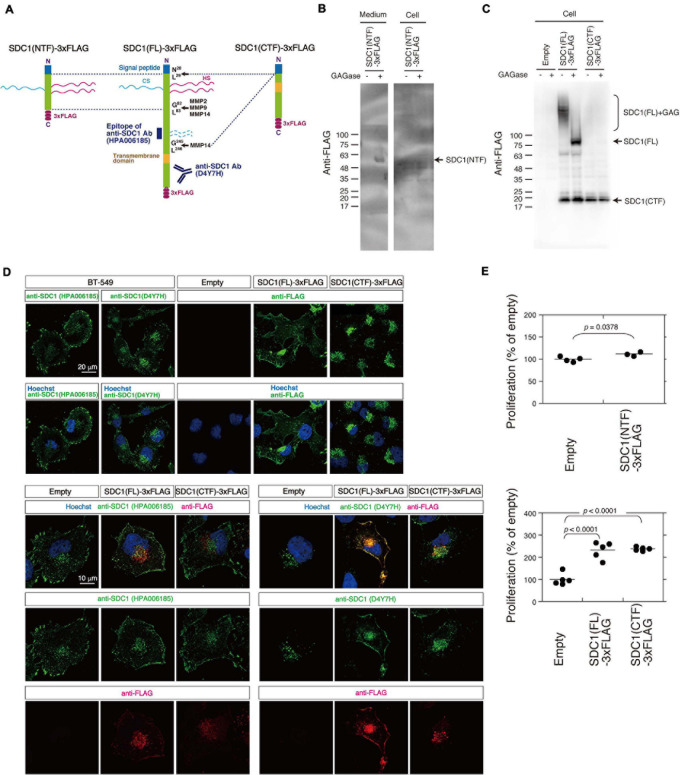
Cellular localization of SDC1(FL)-3xFLAG and SDC1(CTF)-3xFLAG and the effect of SDC1 fragments on cell proliferation after cleavage of MMPs. **(A)** SDC1(FL)-3xFLAG, SDC1(NTF)-3xFLAG, and SDC1(CTF)-3xFLAG were schematically illustrated. Recognition sites of anti-SDC1 antibodies used in this study are shown. **(B)** The Expression of SDC1(NTF)-3xFLAG was confirmed by immunoblotting. Conditioned medium and cell lysate was digested with (+) or without (−) GAGase (the mixture of Chase ABC, HSase, and Hepase). SDC1(NTF)-3xFLAG was detected in medium digested with GAGase. **(C)** The expression of SDC1(FL)-3xFLAG or SDC1(CTF)-3xFLAG in stable clones of BT-549 cells overexpressing SDC1(FL)-3xFLAG and SDC1(CTF)-3xFLAG was confirmed by immunoblotting. Each cell lysate was digested with (+) or without (−) GAGase (the mixture of chondroitinase ABC, heparitinase, and heparinase). SDC1(FL)-3xFLAG modified with GAG chains is represented by “SDC1(FL) + GAG.” BT-549 cells stably expressing the empty vector p3xFLAG-CMV14 is represented as “empty.” **(D)** Expression pattern of exogenously expressed SDC1(FL)-3xFLAG and SDC1(CTF)-3xFLAG was compared with that of endogenous SDC1 by immunofluorescence method using anti-SDC1 antibodies (HPA00618 and D4Y7H) and anti-FLAG antibody. **(E)** Proliferation of BT-549 cells overexpressing the empty vector (*n* = 4) and SDC1(NTF)-3xFLAG (*n* = 3), or proliferation of BT-549 cells overexpressing the empty vector (*n* = 5), SDC1(FL)-3xFLAG (*n* = 5), and SDC1(CTF)-3xFLAG (*n* = 5) was measured.

We tested the possibility that the N- and C-terminal fragment of SDC1 generated by MMPs controls the proliferation of BT-549 cells through the SUMO modification of AKT1. SDC1 is degraded into the N-terminal extracellular fragment (ectodomain) and C-terminal cytoplasmic fragment by MMPs (Figure 4A). We established BT-549 cells stably expressing either the full-length [SDC1(FL)], the N-terminal fragment [SDC1(NTF)], or the C-terminal fragment of SDC1 tagged with FLAG [SDC1(CTF)]. Cell lysates or conditioned medium prepared from the SDC1(FL)-, SDC1(NTF)-, or SDC1(CTF)-expressing cells was analyzed by immunoblotting using the anti-FLAG antibody. SDC1(NTF) was expressed and secreted into the medium (Figure 4B). SDC1(NTF) and SDC1(FL) were expressed as proteoglycans bearing GAG chains. In addition, SDC1(CTF) was detected because of the proteolytic processing of SDC1(FL) (Figure 4C). In the SDC1(CTF)-expressing cells, the C-terminal fragment of SDC1 was detected (Figure 4C).

A previous study suggests that tagging the C-terminus leads to the mislocalization of SDC1 to the apical surface in polarized epithelial cells (Maday et al., 2008). Thus, we next examined the cellular localization of SDC1(FL) and SDC1(CTF) (Figure 4D). Two types of antibodies against SDC1 were used, and their specificities are shown in Figure 4A. Anti-SDC1 antibody (HPA00618) recognizes the N-terminal side of a MMP14 cleavage site (Gly^245^-Leu^246^), while anti-SDC1 antibody (D4YH) binds to the C-terminal side of the transmembrane region. Endogenous SDC1 was localized on the cell surface and in the perinuclear region in BT-549 cells. Localization of SDC1 in the perinuclear region was visualized clearly by anti-SDC1 antibody (D4YH) (Figure 4D), suggesting that the processed C-terminal fragments of SDC1 is localized in the perinuclear region. Cellular localization of exogenously expressed SDC1(FL) and SDC1(CTF) was examined using anti-FLAG antibody. SDC1(FL) was localized at the cell surface and the perinuclear region in a similar pattern to endogenous SDC1, while SDC1(CTF) was localized in the perinuclear region. Double staining with anti-SDC1 antibody and anti-FLAG antibody indicated that the localization of SDC1(FL) was barely affected by the C-terminal FLAG tag. In addition, the localization pattern of SDC1(CTF) visualized by anti-FLAG antibody was merged with the staining pattern obtained using anti-SDC1 antibody (D4Y7H).

**FIGURE 5:**
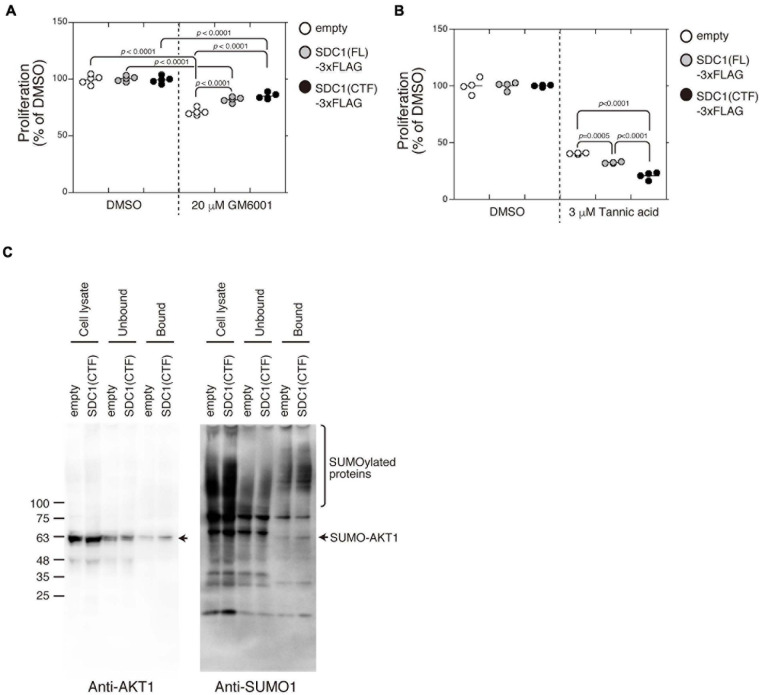
Enhancement of cell proliferation through the SUMOylation of AKT1 upregulated by the expression of the C-terminal fragment of SDC1. **(A)** The effect of GM6001 on the proliferation of BT-549 cells overexpressing the empty vector (*n* = 5), SDC1(FL)-3xFLAG (*n* = 5), and SDC1(CTF)-3xFLAG (*n* = 5) was examined. **(B)** The effect of tannic acid on the proliferation of BT-549 cells overexpressing the empty vector (*n* = 4), SDC1(FL)-3xFLAG (*n* = 4), and SDC1(CTF)-3xFLAG (*n* = 4) was examined. **(C)** SUMOylated proteins in BT-549 cells overexpressing the empty vector and SDC1(CTF)-3xFLAG were captured using the SUMO-QAPTURE-T kit, and subject to immunoblotting using the anti-Akt1 and anti-SUMO1 antibodies.

Proliferation of BT-549 cells was significantly increased by the expression of either SDC1(FL), SDC1(NTF), or SDC1(CTF) (Figure 4E). Cell proliferation is more affected by the expression of SDC1(CTF) than SDC1(NTF). Thus, we examined the effect of SDC1(CTF) on cell proliferation in this study. The inhibitory effect of GM6001 treatment on proliferation was weakened by the expression of either SDC1(FL) or SDC1(CTF) (Figure 5A). Furthermore, cell proliferation was relatively sensitive to tannic acid due to the expression of SDC1(CTF) (Figure 5B). These results suggest that SUMOylation has a greater effect on growth signaling of SDC1(CTF)-expressing BT-549 cells compared with empty vector-expressing BT-549 cells. We next determined the levels of SUMOylated proteins using a commercial SUMO-QAPTURE-T kit (Figure 5C). The SUMOylated protein-enriched bound fraction isolated from cells expressing SDC1(CTF) contained much more AKT1 and SUMOylated proteins than the empty vector-expressing BT-549 cells. These results indicate that the C-terminal fragment of SDC1 regulates the proliferation of BT-549 cells mediated by SUMO modification of AKT1.

## Discussion

### Role of MMPs in Breast Cancer

**FIGURE 6:**
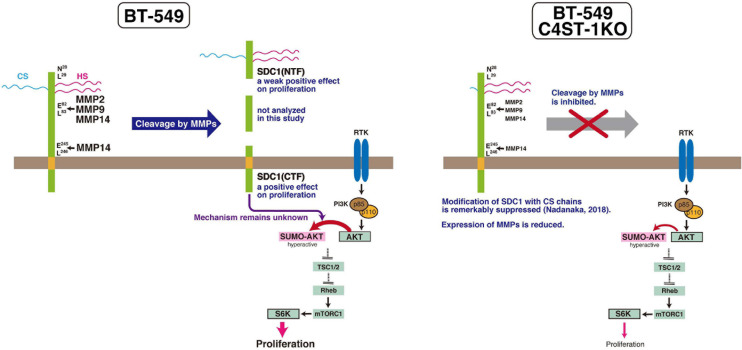
C4ST-1 controls BT-549 cell proliferation by regulating the cleavage of SDC1 by MMPs. SDC1 expressed at the cell surface is cleaved by MMPs, and the N-terminal and the C-terminal fragments of SDC1 are generated. Both fragments, SDC1(NTF) and SDC1(CTF), have a positive effect on cell proliferation, and cell proliferation is more affected by SDC1(CTF) than by SDC1(NTF). SDC1(CTF) is involved in SUMOylation of AKT1, although the mechanism underlying SUMOylation by SDC1(CTF) remains unclear. SUMOylation of AKT1 enhances proliferation through S6 kinase signaling pathway. In C4ST-1KO cells, the cleavage of SDC1 by MMPs is suppressed because of reduced expression of MMPs. SUMOylation of AKT1 is inhibited because of decreased SDC1(CTF), and proliferation is slowed down.

MMPs are members of the metzincin group of zinc-dependent endopeptidases that are responsible for degrading and remodeling the extracellular matrix during cancer progression. We previously showed that CS produced by C4ST-1 induces the expression of MMP9 mediated via the N-cadherin/β-catenin pathway and upregulates the invasive activity of BT-549 cells. MMP-2, MMP-9, and MMP-14, which are involved in the cleavage of SDC1, facilitate cell invasion and metastasis. In addition, these MMPs may regulate tumor proliferation by degradation of the extracellular matrix and basement membrane. MMPs can free growth factors from attachment to matrix components or the cell surface from where they can act on receptors. For example, MMP9 degrades insulin-like growth factor binding protein, which prevents insulin-like growth factor (IGF) from acting on its receptors. Thus, MMP9 can liberate active IGF and induce the activation of the IGF receptor pathway to promote tumor growth (Park et al., 2015). Graphical abstract of this study is shown in Figure 6. We showed that C4ST-1 deficiency could suppress cell proliferation and cleavage of SDC1 by MMPs through controlling the expression of MMPs. Although we previously showed that loss of C4ST-1 remarkably suppresses the modification of SDC1 with CS chains (Nadanaka et al., 2018), it remains unclear whether this alteration of glycosylation makes hard to cleave SDC1 using MMPs. In addition, we examined the mechanism underlying SDC1 cleavage-dependent cell proliferation. We found that the C-terminal fragment of SDC1 generated by MMPs enhanced cell proliferation through SUMOylation of AKT1. However, some issues need to be addressed. We demonstrated cell proliferation-controlling system taking advantage of SDC1 cleavage by MMPs only in a BT-549 cell line. Further studies are needed to examine whether proliferation of basal-like breast cancer cells is ubiquitously regulated by this system. In addition, it remains unclear how the C-terminal fragment of SDC1 controls SUMOylation of AKT1.

In this study, we focused on the cleavage of SDC1 to examine the mechanism underlying C4ST-1-regulated cell proliferation. However, CS chains produced by C4ST-1 may play important roles in controlling proliferation signaling. The biological importance of specific sulfation of CS chains has been reported. A gene trap mutation of *C4ST-1* in mice causes severe chondrodysplasia associated with alterations of growth factor signaling including TGF-β signaling and BMP signaling (Klüppel et al., 2005). In addition, we have previously analyzed CS chains produced in C4ST-1KO cells (Nadanaka et al., 2018). The amount of CS chains decreased by almost half in C4ST-1KO cells compared to that in BT-549 cells. The 4-*O*-sulfated structures were decreased while the 6-*O*-sulfated structures were increased in C4ST-1KO cells, compared with BT-549 cells. Thus, we cannot exclude the possibility that decreased growth factor signaling in C4ST-1 KO cells retards cell proliferation.

The 4-*O*-sulfation of CS is catalyzed by C4ST-1, C4ST-2 (Gene symbol: CHST12), and C4ST-3 (Gene symbol: CHST13) (Mikami and Kitagawa, 2013). However, C4ST-2 and C4ST-3 cannot compensate for the loss of C4ST-1 because a deficiency or experimental knockdown of C4ST-1 results in a drastic decrease in cellular and whole-body level of CS (Klüppel et al., 2005; Uyama et al., 2006). C4ST-1 plays a distinct regulatory role not only in CS 4-*O*-sulfation but also in the amount of CS synthesis (Klüppel et al., 2005; Uyama et al., 2006). Consistent with this finding, we indicated that none of the CS chains are attached to a SDC1 core protein in C4ST-1KO cells (Nadanaka et al., 2018). Thus, it is suggested that C4ST-1 contributes greatly to the biosynthesis of CS chains involved in cell proliferation.

### Role of SDC1 Proteolysis in Tumor Progression

SDC1 is predominantly known as a cell surface proteoglycan that acts as a co-receptor for a variety of growth factor receptors. However, SDC1 is not always localized on the cell surface. It undergoes proteolytic cleavage in a process known as shedding, and releases its extracellular domain (ectodomain) and cytoplasmic domain. Ectodomain shedding reduces the number of membrane-bound type SDC1, serving as a surface receptor, thus downregulating signal transduction. The shed ectodomain contains intact HS chains, allowing the ectodomain to retain its ability to bind growth factors and other extracellular matrix components. Thus, a soluble ectodomain can compete with membrane-bound SDC1 for extracellular ligands (Steinfeld et al., 1996). Nikolova et al. (2009) showed that the overexpression of full-length SDC1 increases cell proliferation of the human breast cancer cell line MCF-7, whereas the shed ectodomain of SDC1 decreases proliferation. In addition, they indicated that the invasion of MCF-7 cells is promoted by the expression of the soluble ectodomain of SDC1. Moreover, shed SDC1 from stromal fibroblasts has been reported to be essential for breast carcinoma angiogenesis (Maeda et al., 2006) and growth of breast cancer cells was stimulated by shed SDC1 from stromal fibroblasts via the activation of FGF-2 (Su et al., 2007, 2008). Chemotherapy-induced SDC1 shedding is mediated by disintegrin and metalloproteinases (ADAMs), and shed SDC1 promotes growth factor signaling (Ramani and Sanderson, 2014). Furthermore, SDC1 shedding correlates with the enhancement of both vascular endothelial growth factor and hepatocyte growth factor signaling and affects angiogenesis (Purushothaman et al., 2010; Ramani et al., 2011). Taken together, the shed ectodomain of SDC1 acts on cancer cells and has powerful effects on their behavior in a context-dependent manner.

Once SDC1 is shed from the cell surface by ADAM17, the remaining transmembrane C-terminal fragment undergoes intramembrane proteolysis by γ-secretase and is degraded by the proteasome in A549 lung cancer cells (Pasqualon et al., 2015a). The C-terminal transmembrane fragment mediates SDC1-dependent functions in cell proliferation, migration, invasion, and metastasis formation (Pasqualon et al., 2015b), whereas the cytoplasmic C-terminal fragment of SDC1 generated by γ-secretase inhibits SDC1-dependent tumor cell migration and invasion by increasing the phosphorylation of Src and focal adhesion kinase (Pasqualon et al., 2015a). In addition, the transmembrane C-terminal fragment does not affect the proliferation of A549 lung cancer cells in the presence of endogenous SDC1 (Pasqualon et al., 2015b). In this study, we examined the effect of the transmembrane C-terminal fragment cleaved at Gly^245^-Leu^246^ by MMP14 on cell proliferation. The expression of this fragment enhances the cell proliferation of BT-549 human breast cancer cells even in the presence of endogenous SDC1. This discrepancy may be explained as follows: ADAM17 cleaves at Val^252^-Leu^253^ of SDC1, releasing the transmembrane C-terminal fragment of SDC1 (amino acid position 253–310), whereas MMP14 produces the transmembrane C-terminal fragment of SDC1 (amino acid position 246–310). Thus, the transmembrane C-terminal fragment of SDC1 (amino acid position 246–310) may be processed in a distinct mechanism from the transmembrane C-terminal fragment of SDC1 (amino acid position 253–310). The two types of cytoplasmic C-terminal fragments of SDC1 generated in a distinct manner may have different effects on cell proliferation. Further studies are needed to explain the distinct functions of these transmembrane C-terminal fragments of SDC1.

### Significance of SUMOylation Required to Maintain the Basal-Like Cancer Subtype

SUMOylation involves the post-translational modification of proteins through the covalent attachment of SUMO proteins to lysine in target proteins, and affects several aspects of oncogenesis and cancer progression (Bettermann et al., 2012). There is a growing body of evidence that the SUMOylation of transcription factors affects transcriptional regulation (Gill, 2003). In addition, the SUMOylation of the serine threonine kinase AKT1 regulates cell proliferation by controlling AKT1 phosphorylation and activity (De La Cruz-Herrera et al., 2015; Lin et al., 2016). Lin et al. (2016) suggested that global SUMOylation occurs through the phosphorylation of Ubc9 and SUMO1 directly mediated by AKT1. Thus, AKT1 is suggested to play an important role in the SUMOylation of a large number of substrate proteins in the cells. Furthermore, the SUMOylation of TFAP2A transcription factor is involved in the transition from the luminal subtype to the basal-like subtype of breast cancer (Bogachek et al., 2014). Because the SUMOylation of TFAP2A blocks its ability to induce the expression of luminal genes, inhibition of the SUMO pathway induces a mesenchymal-to-epithelial transition, characterized by the repression of basal-associated gene expression and induction of luminal-associated genes. These findings indicate that the cleavage of SDC1 controls the SUMOylation of transcription factors associated with the maintenance of the basal-like cancer phenotypes through the SUMOylation of AKT1.

In this study, we found that the C-terminal fragment of SDC1 generated by MMPs could be involved in the SUMOylation of AKT1 in BT-549 cells and that the SDC1-dependent SUMO pathway enhances cell proliferation. Braun et al. (2012) reported that the SDC1 cytoplasmic domain interacts with the ubiquitin and SUMO-1 E3 ligase Topors. They discussed the role of the binding of SDC1 to Topors. Topors is a growth promoter for arterial smooth muscle cells, and SDC1 prevents its growth-promoting activities by inhibiting the ligase activity of Topors by directly binding to Topors. Although the findings reported by Braun et al. are inconsistent with our results, they have not examined whether the binding of SDC1 to Topors affects the SUMOylation pathway in the cells. Recently, Topors has been reported to function as a regulator of chromatin structure (Ji et al., 2020). In addition, the full-length form of SDC1 can translocate to the nucleus, although the complete route of full-length SDC1 internalization has not been elucidated (Brockstedt et al., 2002). Thus, the C-terminal fragment of SDC1 is suggested to induce SUMOylation by a Topors-independent mechanism.

The C-terminal domain of syndecan may serve as a platform to conjugate SUMO to proteins. A membrane-associated guanylate kinase protein, CASK, which is SUMOylated in neurons, has been reported to be a binding partner of syndecan 2 (Chao et al., 2008). Although it has not been investigated whether syndecan 2 is needed for SUMOylation of CASK, both SUMOylated CASK and syndecan 2 contribute to spine maturation. Thus, it is implicated that SUMOylation of substrate proteins such as CASK can be facilitated by forming a complex with syndecan. Moreover, a SUMO-specific isopeptidase, SENP2, has been reported to bind to intracellular membranes where it interacts with membrane-associated proteins (Odeh et al., 2018). As shown in Figure 4D, the C-terminal fragment of SDC1 is localized in the perinuclear region, probably in the Golgi. Membrane-associated proteins such as the C-terminal fragment of SDC1 may have the potential to regulate SUMOylation through binding to SUMO enzymes, although the mechanism by which SUMOylation is regulated at the membrane is largely unexplored.

## Data Availability Statement

The raw data supporting the conclusions of this article will be made available by the authors, without undue reservation.

## Author Contributions

SN and YB performed the research and analyzed the data. SN and HK wrote the manuscript, conceived, and designed the study. HK coordinated the study. All authors reviewed the results and approved the final version of the manuscript.

## Conflict of Interest

The authors declare that the research was conducted in the absence of any commercial or financial relationships that could be construed as a potential conflict of interest.
