# Supplementary material for: Cleavage of Syndecan-1 Promotes the Proliferation of the Basal-Like Breast Cancer Cell Line BT-549 Via Akt SUMOylation
Source: Front Cell Dev Biol. 2021 May 25;9:659428. doi: 10.3389/fcell.2021.659428 (PMC8185021; doi:10.3389/fcell.2021.659428)
Supplement: Supplementary file 1 [file Data_Sheet_1.docx]

***Supplementary Material***

**Supplementary Table S1 List of the antibodies used in this study**

| Antibody | Isotype | Source |
| --- | --- | --- |
| ERK1/2 | Rabbit IgG | Cell Signaling, #9120 |
| Phospho-ERK1/2 | Rabbit IgG | Cell Signaling, #9101 |
| PI3K p85 | Rabbit IgG | Cell Signaling, #4257 (Clone 19H8) |
| Phospho-PI3K p85 (Tyr458)/p55 | Rabbit IgG | Cell Signaling, #4228 |
| STAT3 | Rabbit IgG | Cell Signaling, #9132 |
| Phospho-STAT3(Tyr705) | Rabbit IgG | Cell Signaling, #9131 |
| S6K | Rabbit IgG | Cell Signaling, #2708 (Clone 49D7) |
| Phospho-S6K(Thr389) | Rabbit IgG | Cell Signaling, #9234 (Clone 108D2) |
| SUMO-1 | Mouse IgG_1_ | MBL, #M113-3 (Clone 5B12) |
|  | Rabbit IgG | Enzo, #BML-PW0505A-0010 |
| Syndecan-1 | Rabbit IgG | ATLAS ANTIBODIES, HPA006185 |
|  | Rabbit IgG | Cell Signaling, #12922 (Clone D4Y7H) |
| MMP2 | Rabbit IgG | Proteintech, 10373-2-AP |
| MMP9 | Rabbit IgG | Proteintech, 10375-2-AP |
| MMP14 | Rabbit IgG | Proteintech, 14552-1-AP |

**Supplementary Table S2 List of the primers of real-time PCR used in this study**

| Gene Name | 5’-primer | 3’-primer |
| --- | --- | --- |
| hG3PDH  hMMP2  hMMP7  hMMP9  hMMP14  hCCND1 | 5’-ATGGGTGTGAACCATGAGAAGTA-3’  5’-TCTCCTGACATTGACCTTGGC-3’  5’- TGAGCTACAGTGGGAACAGG-3’  5’-TTGACAGCGACAAGAAGTGG-3’  5’-TTGGACTGTCAGGAATGAGG-3’  5’-ATCTGATCGGGGGCGTAGCA-3’ | 5’-GGCAGTGATGGCATGGAC-3’  5’- CAAGGTGCTGGCTGAGTAGATC-3’  5’-TCATCGAAGTGAGCATCTCC-3’  5’-GCCATTCACGTCGTCCTTAT-3’  5’-GCAGCACAAAATTCTCCGTG-3’  5’-ACGCCGTGGTGGCACGTAAG-3’ |
